# Third-order transport coefficients for localised and delocalised charged-particle transport

**DOI:** 10.1038/s41598-018-19711-5

**Published:** 2018-02-02

**Authors:** Peter W. Stokes, Ilija Simonović, Bronson Philippa, Daniel Cocks, Saša Dujko, Ronald D. White

**Affiliations:** 1 College of Science and Engineering, James Cook University, Townsville, QLD 4811 Australia; 2 Institute of Physics, University of Belgrade, PO Box 68, 11080 Zemun Belgrade, Serbia; 3 College of Science and Engineering, James Cook University, Cairns, QLD 4870 Australia

## Abstract

We derive third-order transport coefficients of skewness for a phase-space kinetic model that considers the processes of scattering collisions, trapping, detrapping and recombination losses. The resulting expression for the skewness tensor provides an extension to Fick’s law which is in turn applied to yield a corresponding generalised advection-diffusion-skewness equation. A physical interpretation of trap-induced skewness is presented and used to describe an observed negative skewness due to traps. A relationship between skewness, diffusion, mobility and temperature is formed by analogy with Einstein’s relation. Fractional transport is explored and its effects on the flux transport coefficients are also outlined.

## Introduction

Very little data regarding third-order transport coefficients (the skewness tensor) can be found in the literature. This is understandable, since they have not been included in the interpretations of traditional swarm experiments. There is, however, a growing interest regarding these transport coefficients, partially due to estimations that third-order transport coefficients could be measured in the present or near future^1,2^. It is also considered that third-order transport coefficients would be very useful, in combination with transport coefficients of a lower order, for determination of cross section sets, by means of inverse swarm procedure^1,2^. Third-order transport coefficients are also required for the conversion of the hydrodynamic transport coefficients into transport data measured in steady state Townsend and arrival time spectra experiments^3,4^. The skewness tensor can also be employed in fluid models of discharges, by pairing a generalised diffusion equation, which includes the contributions of the third-order transport coefficients, with Poisson’s equation. This could be particularly important for discharges where ions play an important role^5^, or in situations where the hydrodynamic approximation is at the limit of applicability, as in the presence of sources and sinks of particles or in the close vicinity of physical boundaries.

**Figure 1:**
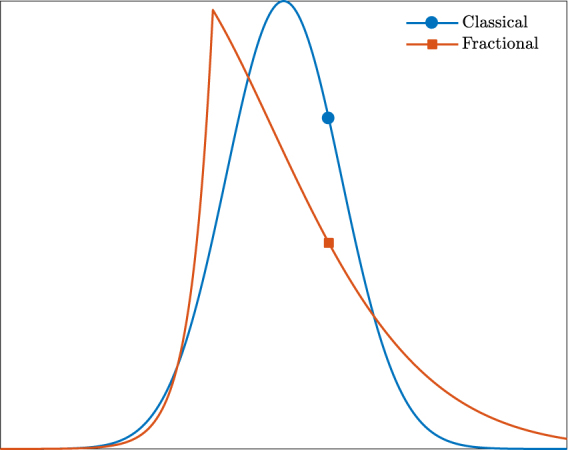
Skewed solution of the Caputo fractional advection-diffusion equation alongside the corresponding Gaussian solution of the classical advection-diffusion equation. Both pulses have evolved from an impulse initial condition. The cusp in the fractional solution denotes the location of this initial impulse.

In this study, we are concerned with the form of the skewness tensor for charged-particle transport in the presence of trapped (localised) states. In particular, we are interested in the scenario where transport is dispersive. Dispersive transport is characterised by a mean squared displacement that increases sublinearly with time^6^. Due to this non-integer power-law dependence, we refer to dispersive transport as fractional transport throughout this study. Some examples of fractional transport include the trapping of charge carriers in local imperfections in semiconductors^7–11^ and both electron^12–14^ and positronium^15–17^ trapping in bubble states within liquids. Third-order transport coefficients are expected to be more sensitive to the influence of non-conservative collisions than those of lower order, suggesting that the presence of such trapped states would significantly influence the skewness tensor. Indeed, skewness and other higher order transport coefficients are used to characterise fractional transport in a variety of contexts, including transport in biological cells^18–21^. Consider also Fig. 1, which plots the solution of the Caputo fractional advection-diffusion equation, a common model for fractional transport^6^. This solution exhibits a large skewness in comparison to the accompanying Gaussian solution of the corresponding classical advection-diffusion equation.

In the following, we describe charged particle transport using a full phase-space kinetic model as defined by a generalised Boltzmann equation with a corresponding trapping and detrapping operator. In our previous papers^22–24^, we introduced and studied such a generalised Boltzmann equation, deriving lower-order transport coefficients (up to diffusion) and generalisations of the Einstein relation. We will extend the results of these papers to determine the skewness tensor. Calculations of the skewness tensor for the Boltzmann equation have been performed previously by a number of authors^2,25–28^. We will use these earlier studies to confirm the structure of the skewness tensor and to benchmark our results in the trap-free case.

In Sec. 2 of this study, we outline a general phase-space kinetic model^23^ for charged-particle transport via localised and delocalised states. This model is capable of describing both normal and fractional transport. We follow in Sec. 3 with a derivation of the flux transport coefficients for this model up to third order. Sec. 4 explores the structure of these transport coefficients and their symmetries under parity transformation. The transport coefficients are used to extend Fick’s law, which leads to a generalised advection-diffusion-skewness equation, presented in Sec. 5. In this section, we also provide a physical interpretation of trap-induced skewness. By analogy with Einstein’s relation, Sec. 6 provides a relation between skewness, diffusion, mobility and temperature. Sec. 7 looks at the case of fractional transport and its effects on the flux transport coefficients. Finally, Sec. 8 lists conclusions along with possible avenues for future work.

## Phase-space kinetic model

We previously reported^22–24^ the development of a phase-space kinetic model wherein charged particles scatter due to collisions, enter and leave traps and undergo recombination. In this model, free particles are described by a phase-space distribution function *f*(*t*, **r**, **v**), defined by the generalised Boltzmann equation

1 $$\begin{array}{ccl}(\frac{{\rm{\partial }}}{{\rm{\partial }}t}+{\bf{v}}\cdot \frac{{\rm{\partial }}}{{\rm{\partial }}{\bf{r}}}+\frac{e{\bf{E}}}{m}\cdot \frac{{\rm{\partial }}}{{\rm{\partial }}{\bf{v}}})\,f(t,{\bf{r}},{\bf{v}}) & = & -{\nu }_{{\rm{c}}{\rm{o}}{\rm{l}}{\rm{l}}}[f(t,{\bf{r}},{\bf{v}})-n(t,{\bf{r}})w({T}_{{\rm{c}}{\rm{o}}{\rm{l}}{\rm{l}}},v)]\\  &  & -{\nu }_{{\rm{t}}{\rm{r}}{\rm{a}}{\rm{p}}}[f(t,{\bf{r}},{\bf{v}})-{\rm{\Phi }}(t)\ast \,n(t,{\bf{r}})w({T}_{{\rm{d}}{\rm{e}}{\rm{t}}{\rm{r}}{\rm{a}}{\rm{p}}},v)]\\  &  & -{\nu }_{{\rm{l}}{\rm{o}}{\rm{s}}{\rm{s}}}^{({\rm{f}}{\rm{r}}{\rm{e}}{\rm{e}})}f(t,{\bf{r}},{\bf{v}}),\end{array}$$

where **E** is the applied electric field and particles have charge *e*, mass *m* and number density *n*(*t*, **r**) ≡ ∫ d**v***f* (*t*, **r**, **v**).

Here, collisions, trapping and free particle recombination occur at the constant frequencies *ν*_coll_, *ν*_trap_ and ${\nu }_{{\rm{loss}}}^{({\rm{free}})}$, respectively. For collisions, the Bhatnagar—Gross—Krook (BGK) collision operator^29^ has been used, which scatters particles isotropically according to a Maxwellian velocity distribution of background temperature *T*_coll_. We define the Maxwellian velocity distribution of temperature *T* as

2 $$w(T,v)\equiv {(\frac{m}{2\pi {k}_{{\rm{B}}}T})}^{\frac{3}{2}}\exp (-\frac{m{v}^{2}}{2{k}_{{\rm{B}}}T}),$$

where *k*_B_ is the Boltzmann constant. Similarly, we use the BGK-type operator introduced by Philippa *et al*.^22^ to describe the processes of trapping and detrapping. This operator specifies that particles leave traps with a Maxwellian distribution of velocities of temperature *T*_detrap_ after a delay that is governed by the distribution of trapping times *ϕ*(*t*). That is, trapping events are described mathematically as delayed scattering events. This distribution appears in Eq. (1) through the effective waiting time distribution

3 $${\rm{\Phi }}(t)\equiv {{\rm{e}}}^{-{\nu }_{{\rm{loss}}}^{({\rm{trap}})}t}\varphi (t),$$

that takes into account trapped particle recombination at the frequency ${\nu }_{{\rm{loss}}}^{({\rm{trap}})}$.

## Transport coefficients to third order

By integrating the generalised Boltzmann equation (1) throughout all of velocity space, we find the equation of continuity for the number density *n*(*t*, **r**):

4 $$[\frac{{\rm{\partial }}}{{\rm{\partial }}t}+{\nu }_{{\rm{t}}{\rm{r}}{\rm{a}}{\rm{p}}}(1-{\rm{\Phi }}(t)\ast )+{\nu }_{{\rm{l}}{\rm{o}}{\rm{s}}{\rm{s}}}^{({\rm{f}}{\rm{r}}{\rm{e}}{\rm{e}})}]\,n(t,{\bf{r}})+\frac{{\rm{\partial }}}{{\rm{\partial }}{\bf{r}}}\cdot {\boldsymbol{\Gamma }}(t,{\bf{r}})=0,$$

where the particle flux is

5 $${\boldsymbol{\Gamma }}(t,{\bf{r}})\equiv \int {\rm{d}}{\bf{v}}\,{\bf{v}}f(t,{\bf{r}},{\bf{v}}).$$

In the weak-gradient hydrodynamic regime, physical quantities can be written as an infinite series of spatial gradients of the number density *n*(*t*, **r**)^30,31^. In the case of the flux **Γ**(*t*, **r**), such a density gradient expansion provides a generalisation of Fick’s law:

6 $${\boldsymbol{\Gamma }}={\bf{W}}n-{\bf{D}}\cdot \frac{\partial n}{\partial {\bf{r}}}+{\bf{Q}}:\frac{{\partial }^{2}n}{\partial {\bf{r}}\partial {\bf{r}}}-\cdots ,$$

where **W** is the drift velocity vector, **D** is the rank-2 diffusion tensor and **Q** is the rank-3 skewness tensor. To determine these flux transport coefficients it is simply a matter of writing the solution of the generalised Boltzmann equation (1) itself as a density gradient expansion

7 $$f(t,{\bf{r}},{\bf{v}})={f}^{(0)}({\bf{v}})n+{{\bf{f}}}^{(1)}({\bf{v}})\cdot \frac{\partial n}{\partial {\bf{r}}}+{{\bf{f}}}^{(2)}({\bf{v}}):\frac{{\partial }^{2}n}{\partial {\bf{r}}\partial {\bf{r}}}+\cdots ,$$

and then evaluating the flux using Eq. (5), resulting in the transport coefficients

8 $${\bf{W}}\equiv \int {\rm{d}}{\bf{v}}\,{\bf{v}}{f}^{(0)}({\bf{v}}),$$

9 $${\bf{D}}\equiv \int {\rm{d}}{\bf{v}}\,{\bf{v}}{{\bf{f}}}^{(1)}({\bf{v}}),$$

10 $${\bf{Q}}\equiv \int {\rm{d}}{\bf{v}}\,{\bf{v}}{{\bf{f}}}^{(2)}({\bf{v}}).$$

Substituting the density gradient expansion of *f* (*t*, **r**, **v**) into the Boltzmann equation (1) and equating coefficients of spatial gradients, as done in Sec. IV of ref.^23^, gives the following coefficients

11 $${f}^{(0)}({\bf{s}})=\frac{{\nu }_{{\rm{coll}}}w\,({\alpha }_{{\rm{coll}}},s)+R{\nu }_{{\rm{trap}}}w\,({\alpha }_{{\rm{detrap}}},s)}{{\nu }_{{\rm{coll}}}+R{\nu }_{{\rm{trap}}}+\frac{e{\bf{E}}}{m}\cdot \imath {\rm{s}}},$$

12 $${{\bf{f}}}^{(1)}({\bf{s}})=\frac{{\nu }_{{\rm{trap}}}{{\bf{R}}}^{(1)}w\,({\alpha }_{{\rm{detrap}}},s)+{f}^{(0)}({\bf{s}})({\bf{W}}-{\nu }_{{\rm{trap}}}{{\bf{R}}}^{(1)})-\imath \frac{\partial {f}^{(0)}}{\partial {\bf{s}}}}{{\nu }_{{\rm{coll}}}+R{\nu }_{{\rm{trap}}}+\frac{e{\bf{E}}}{m}\cdot \imath {\bf{s}}},$$

13 $${{\bf{f}}}^{(2)}({\bf{s}})=\frac{{\nu }_{{\rm{trap}}}{{\bf{R}}}^{(2)}w({\alpha }_{{\rm{detrap}}},s)-{f}^{(0)}({\bf{s}})({\bf{D}}+{\nu }_{{\rm{trap}}}{{\bf{R}}}^{(2)})+{{\bf{f}}}^{(1)}({\bf{s}})({\bf{W}}-{\nu }_{{\rm{trap}}}{{\bf{R}}}^{(1)})-\imath \frac{\partial {{\bf{f}}}^{(1)}}{\partial {\bf{s}}}}{{\nu }_{{\rm{coll}}}+R{\nu }_{{\rm{trap}}}+\frac{e{\bf{E}}}{m}\cdot \imath {\bf{s}}},$$

where a Fourier transform has been performed in velocity space, *f* (**s**) ≡ ∫ d**v**e^− ɩɩ**s** ⋅ **v**^*f* (**v**). As in ref.^23^, we have used the density gradient expansion of the concentration of particles leaving traps:

14 $${\rm{\Phi }}(t)\ast n(t,{\bf{r}})=Rn+{{\bf{R}}}^{(1)}\cdot \frac{\partial n}{\partial {\bf{r}}}+{{\bf{R}}}^{(2)}:\frac{{\partial }^{2}n}{\partial {\bf{r}}\partial {\bf{r}}}+\cdots ,$$

the coefficients of which are related to the flux transport coefficients through

15 $${{\bf{R}}}^{(1)}\equiv \frac{R\langle t\rangle }{1+{\nu }_{{\rm{trap}}}R\langle t\rangle }{\bf{W}},$$

16 $${{\bf{R}}}^{(2)}\equiv \frac{R\langle {t}^{2}\rangle }{2{(1+{\nu }_{{\rm{trap}}}R\langle t\rangle )}^{3}}{\bf{W}}{\bf{W}}-\frac{R\langle t\rangle }{1+{\nu }_{{\rm{trap}}}R\langle t\rangle }{\bf{D}},$$

where the time averages are defined

17 $$\langle \eta \,(t)\rangle \equiv \frac{1}{R}{\int }_{0}^{\infty }{\rm{d}}t{\rm{\Phi }}(t)\,{\rm{e}}\,{}^{[{\nu }_{{\rm{loss}}}^{({\rm{free}})}+{\nu }_{{\rm{trap}}}(1-R)]t}\eta (t).$$

Applying this time average to unity results in an implicit definition for the initial coefficient *R*:

18 $$R\equiv {\int }_{0}^{\infty }{\rm{d}}t{\rm{\Phi }}(t){{\rm{e}}}^{[{\nu }_{{\rm{loss}}}^{({\rm{free}})}+{\nu }_{{\rm{trap}}}(1-R)]t}.$$

Thus, for every trapping time distribution *ϕ*(*t*) there corresponds a value of *R*. Some values are tabulated in Appendix A of ref.^23^.

Proceeding to evaluate Eqs (8–10) for the transport coefficients, we find

19 $${\bf{W}}\equiv \frac{e{\bf{E}}}{m{\nu }_{{\rm{eff}}}},$$

20 $${\bf{D}}\equiv \frac{1}{{\nu }_{{\rm{e}}{\rm{f}}{\rm{f}}}}(\frac{{k}_{{\rm{B}}}{T}_{{\rm{e}}{\rm{f}}{\rm{f}}}}{m}{\bf{I}}+\frac{1+2{\nu }_{{\rm{t}}{\rm{r}}{\rm{a}}{\rm{p}}}R\langle t\rangle }{1+{\nu }_{{\rm{t}}{\rm{r}}{\rm{a}}{\rm{p}}}R\langle t\rangle }{\bf{W}}{\bf{W}}),$$

21 $$\begin{array}{ccc}{\rm{Q}} & \equiv  & [1+{(\frac{1+2{\nu }_{{\rm{t}}{\rm{r}}{\rm{a}}{\rm{p}}}R\langle t\rangle }{1+{\nu }_{{\rm{t}}{\rm{r}}{\rm{a}}{\rm{p}}}R\langle t\rangle })}^{2}-\frac{{\nu }_{{\rm{t}}{\rm{r}}{\rm{a}}{\rm{p}}}R\langle {t}^{2}\rangle }{4{(1+{\nu }_{{\rm{t}}{\rm{r}}{\rm{a}}{\rm{p}}}R\langle t\rangle )}^{3}}{\nu }_{{\rm{e}}{\rm{f}}{\rm{f}}}]\frac{2{\bf{W}}{\bf{W}}{\bf{W}}}{{\nu }_{{\rm{e}}{\rm{f}}{\rm{f}}}^{2}}\\  &  & +\frac{1+2{\nu }_{{\rm{t}}{\rm{r}}{\rm{a}}{\rm{p}}}R\langle t\rangle }{1+{\nu }_{{\rm{t}}{\rm{r}}{\rm{a}}{\rm{p}}}R\langle t\rangle }\frac{{k}_{{\rm{B}}}{T}_{{\rm{e}}{\rm{f}}{\rm{f}}}}{m{\nu }_{{\rm{e}}{\rm{f}}{\rm{f}}}^{2}}({\bf{I}}{\bf{W}}+{{\bf{e}}}_{1}{\bf{W}}{{\bf{e}}}_{1}+{{\bf{e}}}_{2}{\bf{W}}{{\bf{e}}}_{2}+{{\bf{e}}}_{3}{\bf{W}}{{\bf{e}}}_{3})\\  &  & +\frac{{\nu }_{{\rm{t}}{\rm{r}}{\rm{a}}{\rm{p}}}R\langle t\rangle }{1+{\nu }_{{\rm{t}}{\rm{r}}{\rm{a}}{\rm{p}}}R\langle t\rangle }\frac{{\nu }_{{\rm{c}}{\rm{o}}{\rm{l}}{\rm{l}}}}{{\nu }_{{\rm{e}}{\rm{f}}{\rm{f}}}}\frac{{k}_{{\rm{B}}}({T}_{{\rm{c}}{\rm{o}}{\rm{l}}{\rm{l}}}-{T}_{{\rm{d}}{\rm{e}}{\rm{t}}{\rm{r}}{\rm{a}}{\rm{p}}})}{m{\nu }_{{\rm{e}}{\rm{f}}{\rm{f}}}}\frac{{\bf{W}}{\bf{I}}}{{\nu }_{{\rm{e}}{\rm{f}}{\rm{f}}}},\end{array}$$

where **e**_1_, **e**_2_ and **e**_3_ are standard orthonormal basis vectors and we have introduced the effective frequency and temperature:

22 $${\nu }_{{\rm{eff}}}\equiv {\nu }_{{\rm{coll}}}+R{\nu }_{{\rm{trap}}},$$

23 $${T}_{{\rm{eff}}}\equiv \frac{{\nu }_{{\rm{coll}}}{T}_{{\rm{coll}}}+R{\nu }_{{\rm{trap}}}{T}_{{\rm{detrap}}}}{{\nu }_{{\rm{coll}}}+R{\nu }_{{\rm{trap}}}}.$$

We confirm that when there are no traps present, *ν*_trap_ = 0, the transport coefficients agree with those of the BGK collision model, previously found by Robson^26^:

24 $${\bf{W}}\equiv \frac{e{\bf{E}}}{m{\nu }_{{\rm{coll}}}},$$

25 $${\bf{D}}\equiv \frac{1}{{\nu }_{{\rm{c}}{\rm{o}}{\rm{l}}{\rm{l}}}}(\frac{{k}_{{\rm{B}}}{T}_{{\rm{c}}{\rm{o}}{\rm{l}}{\rm{l}}}}{m}{\bf{I}}+{\bf{W}}{\bf{W}}),$$

26 $${\bf{Q}}\equiv \frac{1}{{\nu }_{{\rm{c}}{\rm{o}}{\rm{l}}{\rm{l}}}^{2}}\,[\frac{{k}_{{\rm{B}}}{T}_{{\rm{c}}{\rm{o}}{\rm{l}}{\rm{l}}}}{m}({\bf{I}}{\bf{W}}+{{\bf{e}}}_{1}{\bf{W}}{{\bf{e}}}_{1}+{{\bf{e}}}_{2}{\bf{W}}{{\bf{e}}}_{2}+{{\bf{e}}}_{3}{\bf{W}}{{\bf{e}}}_{3})+4{\bf{W}}{\bf{W}}{\bf{W}}].$$

## Structure and Symmetry of Transport Coefficients

If we align the basis vector **e**_3_ parallel to the applied electric field **E**, the transport coefficients (19–21) take on the known tensor structure^2,25,28,30,31^:

27 $${\bf{W}}\equiv [\begin{array}{c}0\\ 0\\ W\end{array}],$$

28 $${\bf{D}}\equiv [\begin{array}{ccc}{D}_{\perp } & 0 & 0\\ 0 & {D}_{\perp } & 0\\ 0 & 0 & {D}_{\parallel }\end{array}]$$

29 $${{\bf{Q}}}_{xab}\equiv [\begin{array}{ccc}0 & 0 & {Q}_{1}\\ 0 & 0 & 0\\ {Q}_{1} & 0 & 0\end{array}],$$

30 $${{\bf{Q}}}_{yab}\equiv [\begin{array}{ccc}0 & 0 & 0\\ 0 & 0 & {Q}_{1}\\ 0 & {Q}_{1} & 0\end{array}],$$

31 $${{\bf{Q}}}_{zab}\equiv [\begin{array}{ccc}{Q}_{2} & 0 & 0\\ 0 & {Q}_{2} & 0\\ 0 & 0 & 2{Q}_{1}+{Q}_{2}+{Q}_{3}\end{array}],$$

where *a*, *b* ∈ {*x*, *y*, *z*}. Here, the drift velocity is defined by the speed

32 $$W\equiv \frac{eE}{m{\nu }_{{\rm{eff}}}},$$

the diffusion coefficient is defined by two components perpendicular and parallel to the field

33 $${D}_{\perp }\equiv \frac{{k}_{{\rm{B}}}{T}_{{\rm{eff}}}}{m{\nu }_{{\rm{eff}}}},$$

34 $${D}_{\parallel }\equiv {D}_{\perp }+\frac{1+2{\nu }_{{\rm{trap}}}R\langle t\rangle }{1+{\nu }_{{\rm{trap}}}R\langle t\rangle }\frac{{W}^{2}}{{\nu }_{{\rm{eff}}}},$$

and the skewness is defined by the three independent components

35 $${Q}_{1}\equiv \frac{1+2{\nu }_{{\rm{trap}}}R\langle t\rangle }{1+{\nu }_{{\rm{trap}}}R\langle t\rangle }\frac{{k}_{{\rm{B}}}{T}_{{\rm{eff}}}}{m{\nu }_{{\rm{eff}}}}\frac{W}{{\nu }_{{\rm{eff}}}},$$

36 $${Q}_{2}\equiv \frac{{\nu }_{{\rm{trap}}}R\langle t\rangle }{1+{\nu }_{{\rm{trap}}}R\langle t\rangle }\frac{{\nu }_{{\rm{coll}}}}{{\nu }_{{\rm{eff}}}}\frac{{k}_{{\rm{B}}}({T}_{{\rm{coll}}}-{T}_{{\rm{detrap}}})}{m{\nu }_{{\rm{eff}}}}\frac{W}{{\nu }_{{\rm{eff}}}},$$

37 $${Q}_{3}\equiv [1+{(\frac{1+2{\nu }_{{\rm{t}}{\rm{r}}{\rm{a}}{\rm{p}}}R\langle t\rangle }{1+{\nu }_{{\rm{t}}{\rm{r}}{\rm{a}}{\rm{p}}}R\langle t\rangle })}^{2}-\frac{{\nu }_{{\rm{t}}{\rm{r}}{\rm{a}}{\rm{p}}}R\langle {t}^{2}\rangle }{4{(1+{\nu }_{{\rm{t}}{\rm{r}}{\rm{a}}{\rm{p}}}R\langle t\rangle )}^{3}}{\nu }_{{\rm{e}}{\rm{f}}{\rm{f}}}]\,\frac{2{W}^{3}}{{\nu }_{{\rm{e}}{\rm{f}}{\rm{f}}}^{2}}.$$

Although this is the case in general, there are situations where the skewness can be defined using fewer than three components. Indeed, this is the case for the BGK model as studied by Robson^26^ where the skewness given by Eq. (26) is defined using only the components *Q*_1_ and *Q*_3_, with *Q*_2_ = 0. The component *Q*_2_ vanishes in this case due to the simple Maxwellian source term used to describe scattered particles. For *Q*_2_ to arise, it is necessary that this source term has some spatial dependence, as occurs for our model through the concentration of particles leaving traps, ${\varphi }(t)\ast n(t,{\bf{r}})$, and its density gradient expansion (14).

Lastly, we also confirm that the symmetry of transport coefficients with respect to the parity transformation **E **→ −**E** depends on the parity of the order of each transport coefficient^25,32^:

38 $${\bf{W}}\to -{\bf{W}},$$

39 $${\bf{D}}\to {\bf{D}},$$

40 $${\bf{Q}}\to -{\bf{Q}}.$$

## Generalised Advection-diffusion-skewness Equation

Using the density gradient expansion (6) for the flux **Γ**(*t*, **r**) up to second spatial order in conjunction with the continuity equation (4) results in the generalised advection-diffusion-skewness equation

41 $$[\frac{{\rm{\partial }}}{{\rm{\partial }}t}+{\nu }_{{\rm{t}}{\rm{r}}{\rm{a}}{\rm{p}}}(1-{\rm{\Phi }}(t)\ast )+{\nu }_{{\rm{l}}{\rm{o}}{\rm{s}}{\rm{s}}}^{({\rm{f}}{\rm{r}}{\rm{e}}{\rm{e}})}]\,n\,(t,{\bf{r}})+{\bf{W}}\cdot \frac{{\rm{\partial }}n}{{\rm{\partial }}{\bf{r}}}-{\bf{D}}:\frac{{{\rm{\partial }}}^{2}n}{{\rm{\partial }}{\bf{r}}{\rm{\partial }}{\bf{r}}}+{\bf{Q}}\vdots \frac{{{\rm{\partial }}}^{3}n}{{\rm{\partial }}{\bf{r}}{\rm{\partial }}{\bf{r}}{\rm{\partial }}{\bf{r}}}=0,$$

valid in the weak-gradient hydrodynamic regime. In Cartesian coordinates (*x*, *y*, *z*) with the electric field **E** aligned in the *z*-direction, the transport coefficients take the form of Eqs (27–31) and the advection-diffusion-skewness equation becomes

42 $$\begin{array}{c}[\frac{{\rm{\partial }}}{{\rm{\partial }}t}+{\nu }_{{\rm{t}}{\rm{r}}{\rm{a}}{\rm{p}}}(1-{\rm{\Phi }}(t)\ast )+{\nu }_{{\rm{l}}{\rm{o}}{\rm{s}}{\rm{s}}}^{({\rm{f}}{\rm{r}}{\rm{e}}{\rm{e}})}]\,n\,(t,x,y,z)+W\frac{{\rm{\partial }}n}{{\rm{\partial }}z}-{D}_{\perp }(\frac{{{\rm{\partial }}}^{2}n}{{\rm{\partial }}{x}^{2}}+\frac{{{\rm{\partial }}}^{2}n}{{\rm{\partial }}{y}^{2}})-{D}_{\parallel }\frac{{{\rm{\partial }}}^{2}n}{{\rm{\partial }}{z}^{2}}\\ \quad \,\,\,+3{Q}_{\perp }(\frac{{{\rm{\partial }}}^{2}}{{\rm{\partial }}{x}^{2}}+\frac{{{\rm{\partial }}}^{2}}{{\rm{\partial }}{y}^{2}})\frac{{\rm{\partial }}n}{{\rm{\partial }}z}+{Q}_{\parallel }\frac{{{\rm{\partial }}}^{3}n}{{\rm{\partial }}{z}^{3}}=0,\end{array}$$

where the skewness manifests as components perpendicular and parallel to the applied field^2,5,28^:

43 $${Q}_{\perp }\equiv \frac{{Q}_{zxx}+{Q}_{xzx}+{Q}_{xxz}}{3},$$

44 $${Q}_{\parallel }\equiv {Q}_{zzz},$$

which in terms of the independent components (35–37) are

45 $${Q}_{\perp }=\frac{2{Q}_{1}+{Q}_{2}}{3},$$

46 $${Q}_{\parallel }=2{Q}_{1}+{Q}_{2}+{Q}_{3}.$$

Written in full, the perpendicular and parallel skewnesses are

47 $$\begin{array}{ccc}{Q}_{\perp } & = & \frac{2{D}_{\perp }W}{3{\nu }_{{\rm{e}}{\rm{f}}{\rm{f}}}}\\  &  & \,+\frac{{\nu }_{{\rm{t}}{\rm{r}}{\rm{a}}{\rm{p}}}R\langle t\rangle }{1+{\nu }_{{\rm{t}}{\rm{r}}{\rm{a}}{\rm{p}}}R\langle t\rangle }\,({D}_{\perp }-\frac{{k}_{{\rm{B}}}{T}_{{\rm{d}}{\rm{e}}{\rm{t}}{\rm{r}}{\rm{a}}{\rm{p}}}}{3m{\nu }_{{\rm{e}}{\rm{f}}{\rm{f}}}})\frac{W}{{\nu }_{{\rm{e}}{\rm{f}}{\rm{f}}}},\end{array}$$

48 $$\begin{array}{ccc}{Q}_{\parallel } & = & 3{Q}_{\perp }+\frac{4{W}^{3}}{{\nu }_{{\rm{e}}{\rm{f}}{\rm{f}}}^{2}}\\  &  & \,+\frac{{\nu }_{{\rm{t}}{\rm{r}}{\rm{a}}{\rm{p}}}R\langle t\rangle }{1+{\nu }_{{\rm{t}}{\rm{r}}{\rm{a}}{\rm{p}}}R\langle t\rangle }\,[6-\frac{2}{1+{\nu }_{{\rm{t}}{\rm{r}}{\rm{a}}{\rm{p}}}R\langle t\rangle }-\frac{{\nu }_{{\rm{e}}{\rm{f}}{\rm{f}}}\langle {t}^{2}\rangle }{2\langle t\rangle {(1+{\nu }_{{\rm{t}}{\rm{r}}{\rm{a}}{\rm{p}}}R\langle t\rangle )}^{2}}]\,\frac{{W}^{3}}{{\nu }_{{\rm{e}}{\rm{f}}{\rm{f}}}^{2}},\end{array}$$

where terms present due to trapping have been grouped separately and the lower-order transport coefficients (32–34) have been used to simplify. An alternative form of the skewness tensor that makes use of these components explicitly is

49 $${\mathop{{\bf{Q}}}\limits^{ \sim }}_{xab}\equiv [\begin{array}{ccc}0 & 0 & 0\\ 0 & 0 & 0\\ 0 & 0 & 0\end{array}],$$

50 $${\mathop{{\bf{Q}}}\limits^{ \sim }}_{yab}\equiv [\begin{array}{ccc}0 & 0 & 0\\ 0 & 0 & 0\\ 0 & 0 & 0\end{array}],$$

51 $${\mathop{{\bf{Q}}}\limits^{ \sim }}_{zab}\equiv [\begin{array}{ccc}3{Q}_{\perp } & 0 & 0\\ 0 & 3{Q}_{\perp } & 0\\ 0 & 0 & {Q}_{\parallel }\end{array}],$$

where *a*, *b* ∈ {*x*, *y*, *z*}. This form was used by Robson^26^ when expressing the BGK model skewness (26) and is valid only when the skewness is triple-contracted with a symmetric tensor, as occurs in the advection-diffusion-skewness equation (41).

**Figure 2:**
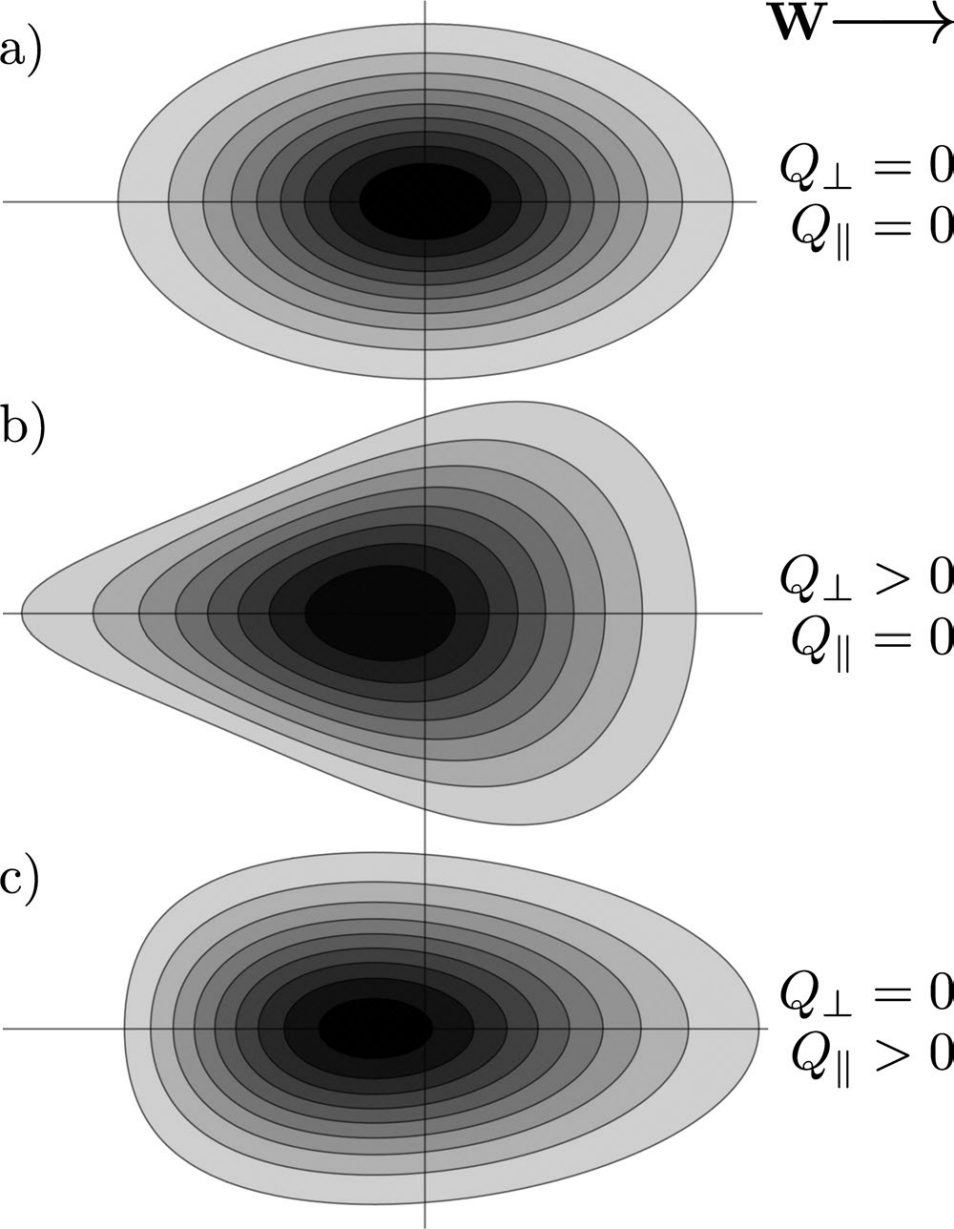
Contours of constant number density as defined by the advection-diffusion-skewness equation (42) with drift velocity **W** and anisotropic diffusion ${D}_{\parallel } > {D}_{\perp } > 0$ for no skewness, (**a**), positive perpendicular skewness, (**b**), and positive parallel skewness, (**c**). Each profile has evolved from an impulse initial condition. As the skewness tensor is odd under parity transformation, Eq. (40), the case of negative skewness can be considered by reflecting the above profiles horizontally across the vertical axis.

To provide some physical intuition regarding the perpendicular and parallel skewness coefficients, *Q*_⊥_ and ${Q}_{\parallel }$, we solve the advection-diffusion-skewness equation (42) for an impulse initial condition and perform contour plots of the resulting pulse in Fig. 2. Figure 2(a) considers the case of no skewness, ${Q}_{\perp }={Q}_{\parallel }=0$, and displays the expected Gaussian solution with elliptical contours due to anisotropic diffusion. Figure 2(b) and (c) consider positive perpendicular and parallel skewnesses, respectively. In both cases, it can be seen that skewness introduces asymmetry in the pulse in the direction of the field. In general, positive skewness can be seen to reduce the spread of particles behind the pulse, while enhancing the spread toward the front of the pulse. In Fig. 2(b) for positive perpendicular skewness, this change in particle spread primarily occurs transverse to the field, resulting in a vaguely triangular pulse profile. In Fig. 2(c) for positive parallel skewness, this change in particle spread occurs longitudinally which, in the language of statistics, results in a distribution with positive skew.

**Figure 3:**
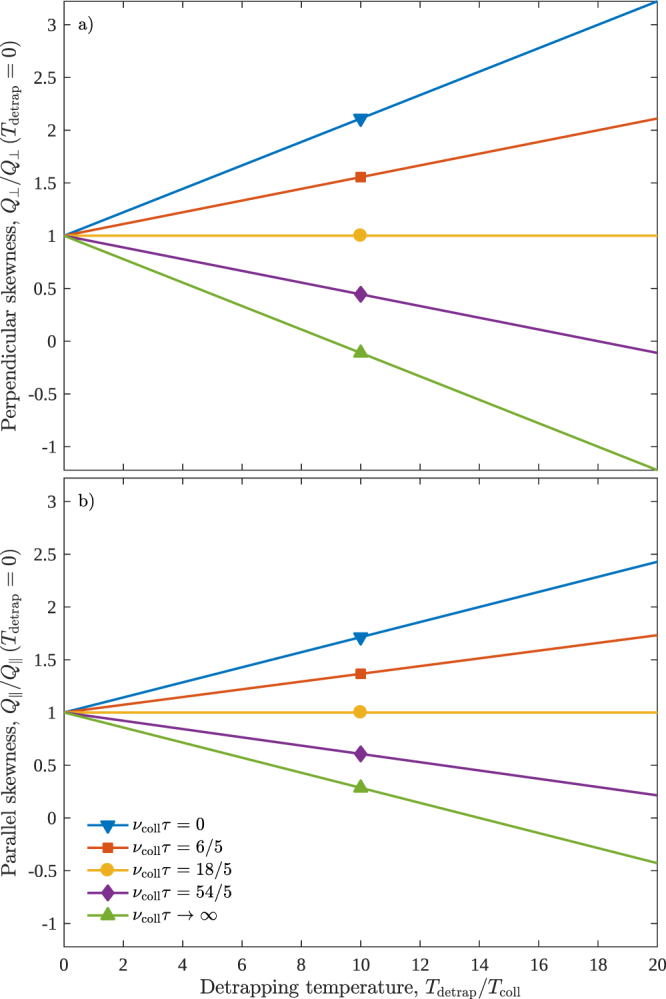
Linear plots of perpendicular and parallel skewness coefficients, *Q*_⊥_ and ${Q}_{\parallel }$, versus the detrapping temperature *T*_detrap_. Here, traps are described by an exponential distribution of trapping times $\varphi (t)=\frac{1}{\tau }\exp (-\frac{t}{\tau })$, and no recombination is considered, ${\nu }_{{\rm{loss}}}^{({\rm{free}})}={\nu }_{{\rm{loss}}}^{({\rm{trap}})}=0$. To perform these plots, we choose a trapping frequency of *ν*_trap_/*ν*_coll_ = 1/9, while (**b**) also requires that we specify a drift velocity **W**, which we choose such that *mW*^2^/*k*_B_*T*_coll_ = 1/4. The gradients in (**b**) are of smaller magnitude than (**a**) due to the greater dependence of the parallel skewness (48) on the drift speed *W* as compared to the perpendicular skewness (47). Thus, as the drift speed decreases, the plots in (**b**) coincide with those in (**a**). When detrapping is instantaneous, *τ* = 0, the skewness gradients are positive, implying that the skewness caused by traps is also positive. As the mean trapping time *τ* increases, the skewness gradients decrease, becoming negative and implying a corresponding negative skewness due to traps. The limiting case of an infinite mean trapping time, *τ* → ∞, corresponds to fractional transport, which is the subject of Sec. 7. We observe from this figure that the skewness coefficients become overall negative when particles leave traps with a sufficiently large temperature *T*_detrap_ after a sufficiently long amount of time *τ*. This observation coincides with the illustration of skewness in Fig. 2 where negative skewness is characterised by an increased particle spread behind the pulse, which we attribute here to particles returning from traps.

In our previous paper^23^, we interpreted the trap-induced anisotropic diffusion present in Eq. (34) as a consequence of the physical separation between trapped particles and free particles moving with the field. In a similar fashion, we can interpret the trap-induced skewness present in the perpendicular and parallel skewness coefficients (47) and (48). To achieve this, we plot the skewness against the detrapping temperature *T*_detrap_ for various mean trapping times in Fig. 3. The resulting plots are linear with gradients that characterise of the type of skewness caused by traps. That is, positive or negative gradients correspond respectively to positive or negative trap-based skewness.

When the mean trapping time is zero, the gradients in Fig. 3 are positive and traps cause positive skewness. This is to be expected as, in this case, trapping and detrapping simply act as an elastic scattering process with a positive skewness akin to Eq. (26) for the BGK collision model. As the mean trapping time increases, the nature of the skewness caused by traps changes, ultimately becoming negative for the parameters considered in Fig. 3. As illustrated in Fig. 2, negative skewness corresponds to an increased spread of particles behind the pulse. We interpret the increased spread here as being due to particles returning from traps. This interpretation implies that the skewness coefficients could become overall negative if particles remain trapped for a sufficient length of time before returning with a sufficiently large temperature. Indeed, these are the conditions for which the skewness coefficients become negative in Fig. 3.

This phenomenon of negative skewness has been observed previously by Petrović *et al*.^5^ in the calculation of the perpendicular skewness of electrons in methane. Only collisions were considered in this study and so trapping is evidently not a necessary condition for negative skewness to occur. However, it should be emphasised that the skewness is strictly positive when collisions are described by the simple BGK collision operator, as is seen in Eq. (26).

## Relating skewness, mobility and temperature

The classical Einstein relation between diffusion, mobility and temperature is^33^

52 $$\frac{{\bf{D}}}{K}=\frac{{k}_{{\rm{B}}}{\bf{T}}}{e},$$

where *K* is the mobility defined as satisfying **W** ≡ *K***E** and **T** is the rank-2 temperature tensor. As seen by Eq. (20) for the diffusion coefficient, the phase-space model described by Eq. (1) has an enhanced diffusivity in the direction of the field due to trapping and detrapping. This enhancement manifests as the following generalised Einstein relation^24^

53 $$\frac{{\bf{D}}}{K}=\frac{{k}_{{\rm{B}}}{\bf{T}}}{e}+\frac{{\nu }_{{\rm{trap}}}R\langle t\rangle }{1+{\nu }_{{\rm{trap}}}R\langle t\rangle }\frac{m{\bf{W}}{\bf{W}}}{e}.$$

By relating the skewness to the temperature tensor though this diffusion coefficient, we find a skewness analogue to the Einstein relation:

54 $$\begin{array}{ccc}{\bf{Q}} & \equiv  & [1-\frac{{\nu }_{{\rm{t}}{\rm{r}}{\rm{a}}{\rm{p}}}R\langle {t}^{2}\rangle }{4{(1+{\nu }_{{\rm{t}}{\rm{r}}{\rm{a}}{\rm{p}}}R\langle t\rangle )}^{3}}{\nu }_{{\rm{e}}{\rm{f}}{\rm{f}}}]\frac{2{\bf{W}}{\bf{W}}{\bf{W}}}{{\nu }_{{\rm{e}}{\rm{f}}{\rm{f}}}^{2}}\\  &  & \,+\frac{1+2{\nu }_{{\rm{t}}{\rm{r}}{\rm{a}}{\rm{p}}}R\langle t\rangle }{1+{\nu }_{{\rm{t}}{\rm{r}}{\rm{a}}{\rm{p}}}R\langle t\rangle }\frac{{\bf{D}}{\bf{W}}+{D}_{\perp }{{\bf{e}}}_{1}{\bf{W}}{{\bf{e}}}_{1}+{D}_{\perp }{{\bf{e}}}_{2}{\bf{W}}{{\bf{e}}}_{2}+{D}_{\parallel }{{\bf{e}}}_{3}{\bf{W}}{{\bf{e}}}_{3}}{{\nu }_{{\rm{e}}{\rm{f}}{\rm{f}}}}\\  &  & \,+\frac{{\nu }_{{\rm{t}}{\rm{r}}{\rm{a}}{\rm{p}}}R\langle t\rangle }{1+{\nu }_{{\rm{t}}{\rm{r}}{\rm{a}}{\rm{p}}}R\langle t\rangle }\frac{{\nu }_{{\rm{c}}{\rm{o}}{\rm{l}}{\rm{l}}}}{{\nu }_{{\rm{e}}{\rm{f}}{\rm{f}}}}\frac{{k}_{{\rm{B}}}({T}_{{\rm{c}}{\rm{o}}{\rm{l}}{\rm{l}}}-{T}_{{\rm{d}}{\rm{e}}{\rm{t}}{\rm{r}}{\rm{a}}{\rm{p}}})}{m{\nu }_{{\rm{e}}{\rm{f}}{\rm{f}}}}\frac{{\bf{W}}{\bf{I}}}{{\nu }_{{\rm{e}}{\rm{f}}{\rm{f}}}}.\end{array}$$

Koutselos^34^ has presented a similar relationship between the skewness tensor and lower-order transport coefficients for the case of the classical Boltzmann equation.

## The case of Fractional Transport

For the phase-space kinetic model described by Eq. (1), fractional transport can occur when the distribution of trapping times has a heavy power-law tail of the form^23^

55 $$\varphi (t)\sim {t}^{-(1+\alpha )}.$$

Note that, as transport here is dispersive in nature, the mean trapping time diverges:

56 $${\int }_{0}^{\infty }{\rm{d}}t\varphi (t)t\to \infty .$$

Consequently, the time averages defined by Eq. (17) also diverge, correspondingly affecting the transport coefficients. Thus, for fractional transport, the transport coefficients (19–21) take on the simpler form^23^

57 $${\bf{W}}=\frac{e{\bf{E}}}{m{\nu }_{{\rm{eff}}}},$$

58 $${\bf{D}}=\frac{1}{{\nu }_{{\rm{e}}{\rm{f}}{\rm{f}}}}(\frac{{k}_{{\rm{B}}}{T}_{{\rm{e}}{\rm{f}}{\rm{f}}}}{m}{\bf{I}}+2{\bf{W}}{\bf{W}}),$$

59 $$\begin{array}{rcl}{\bf{Q}} & = & \frac{2{\bf{W}}{\bf{W}}{\bf{W}}}{{\nu }_{{\rm{eff}}}^{2}}\\  &  & \,+\frac{2({\bf{D}}{\bf{W}}+{D}_{\perp }{{\bf{e}}}_{1}{\bf{W}}{{\bf{e}}}_{1}+{D}_{\perp }{{\bf{e}}}_{2}{\bf{W}}{{\bf{e}}}_{2}+{D}_{\parallel }{{\bf{e}}}_{3}{\bf{W}}{{\bf{e}}}_{3})}{{\nu }_{{\rm{eff}}}}\\  &  & \,+\frac{{\nu }_{{\rm{coll}}}}{{\nu }_{{\rm{eff}}}}\frac{{k}_{{\rm{B}}}({T}_{{\rm{coll}}}-{T}_{{\rm{detrap}}})}{m{\nu }_{{\rm{eff}}}}\frac{{\bf{W}}{\bf{I}}}{{\nu }_{{\rm{eff}}}},\end{array}$$

where the effective frequency is now defined

60 $${\nu }_{{\rm{eff}}}\equiv {\nu }_{{\rm{coll}}}+{\nu }_{{\rm{trap}}}+{\nu }_{{\rm{loss}}}^{({\rm{free}})}.$$

Note that transport coefficients are now independent of the specific choice of the trapping time distribution *ϕ*(*t*), so long as the condition (55) for fractional transport is satisfied.

Knowledge of the skewness coefficient (59), as well as other higher-order transport coefficients, is useful for characterising fractional transport. For example, Norregaard *et al*.^18^ use higher-order moments to analyse the motion of biological particles.

## Conclusion

We have explored the transport coefficients of a phase-space kinetic model (1) for both localised and delocalised transport. In particular, we have considered up to the third-order transport coefficient of skewness bfQ, which takes the form of a rank-3 tensor. The structure of the skewness tensor and its symmetry under parity transformation was found to be in agreement with previous studies. These transport coefficients provide an extension to Fick’s law, Eq. (6), which we used to form a generalised advection-diffusion-skewness equation (41) with a non-local time operator. We observed trap-induced negative skewness and provided a corresponding physical interpretation. In addition, by analogy with Einstein’s relation, the skewness was related to the mobility and temperature through Eq. (54). Lastly, the form of the transport coefficients for the particular case of fractional transport were outlined in Eqs (57–59).

There exist a number of possibilities for future work. The focus of this paper was on constant transport coefficients that define the flux in the hydrodynamic regime as the density gradient expansion (6). Transient transport coefficients and transport coefficients of the bulk were not considered. Ref.^23^ outlines an analytical solution of the kinetic model (1) that could be used to compute such transport coefficients through time-varying velocity and spatial moments of the phase-space distribution function *f* (*t*, **r**, **v**).

Another extension to this work could be to explore what consequences energy-dependent collision, trapping and recombination frequencies have on the skewness. Such a generalisation for Eq. (1) was the focus of ref.^24^. This would allow for the derivation of a skewness analogue of Einstein’s relation that would also take into account the field dependence of mobility^24^. This may also shed light on the recent results of Petrović *et al*.^5^, that suggest a correlation between the energy-dependent phenomenon of negative differential conductivity and skewness.

Lastly, it is important to note that the extension to Fick’s law described in this paper is only useful when an electric field is present. Without an applied field, the drift velocity, skewness and all other odd-ordered transport coefficients would vanish. If we wish to extend Fick’s law in such a situation, we must also consider the kurtosis coefficient, the next even-ordered transport coefficient beyond diffusion. The kurtosis can be found in a straightforward fashion from the rank-3 tensorial coefficient **f**^(3)^(**v**) in the density gradient expansion (7) of the phase-space distribution function *f* (*t*, **r**, **v**), in the same way drift velocity, diffusion and skewness were found using Eqs (8–10).

### Data availability statement

No datasets were generated or analysed during the current study.
